# A Novel Blood Based Mitochondrial Biomarker for Alzheimer’s Disease

**DOI:** 10.1002/alz.090533

**Published:** 2025-01-09

**Authors:** Brittany M Hauger, Keith P Smith, Taylor A. Strope, Ellen Oehmke, Paul J Kueck, Riley E Kemna, Casey S. John, Leonidas Bantis, Jill K Morris, Russell H Swerdlow, Heather M Wilkins

**Affiliations:** ^1^ University of Kansas Medical Center, Kansas City, KS USA; ^2^ University of Kansas Alzheimer's Disease Research Center, Kansas City, KS USA; ^3^ University of Kansas Alzheimer's Disease Research Center, Fairway, KS USA

## Abstract

**Background:**

Alzheimer’s disease (AD) pathology begins decades before clinical onset of dementia. Amyloid beta (Aβ) generally accumulates first in cognitively normal (ND) individuals, with tau and cognitive abnormalities following. AD pathologies have been found to correlate and interact with metabolic and mitochondrial outcomes in studies spanning numerous experimental paradigms. Targeting metabolic and mitochondrial function in clinical trials is an emerging theme for AD and highlights the importance and need for biomarkers which measure mitochondrial function.

**Method:**

20 ND, 20 mild‐cognitive impairment, and 19 Alzheimer’s Disease (AD) subjects were recruited from the KU Alzheimer’s Disease Research Center Clinical Cohort. Blood was collected in ACD tubes. Lymphocytes were isolated using Accuspin tubes, histopaque 1077, and centrifugation. Lymphocytes were stained with MitoTracker/Annexin V, MitoSox/Hoechst, and TMRE/Hoechst. Fluorescent cells were quantified using flow cytometry and values normalized to Hoechst. Fluorescent measures were completed within 30 hours of blood draw. The mitochondrial health index (MHI) algorithm was calculated. Plasma NfL, pTau181, Aβ, and GFAP were determined using SIMOA. APOE genotypes were determined using a Taqman allelic discrimination assay.

**Result:**

The MHI biomarker was significantly reduced in AD subjects and tracked with mini mental state exam (MMSE) scores. APOE ε4 carriers had reduced MHI regardless of diagnosis. Plasma GFAP, NfL, and pTau181 were increased in AD subjects. ROC analysis for individual biomarkers for ND/AD: Aβ 0.6658, GFAP 0.7194, NfL 0.7194, pTau181 0.7796, MHI 0.8028. ROC analysis for individual biomarkers for ND/MCI: Aβ 0.6700, GFAP 0.5737, NfL 0.5368, pTau181 0.7105, MHI 0.6025. ROC analysis for individual biomarkers for MCI/AD: Aβ 0.5632, GFAP 0.5965, NfL 0.6462, pTau181 0.5078, MHI 0.8139.

**Conclusion:**

The MHI biomarker is a blood‐based biomarker with reasonable discrimination for AD diagnosis. Further studies are needed to fully understand how these potential biomarkers associate with disease progression and vary over time. However, mitochondrial biomarkers are urgently needed to assess therapeutic validity in upcoming clinical trials.

